# Comparative Study on the Impact Wedge-Peel Performance of Epoxy-Based Structural Adhesives Modified with Different Toughening Agents

**DOI:** 10.3390/polym12071549

**Published:** 2020-07-13

**Authors:** Gyeong-Seok Chae, Hee-Woong Park, Jung-Hyun Lee, Seunghan Shin

**Affiliations:** 1 Green Chemistry & Materials Group, Korea Institute of Industrial Technology (KITECH), Cheonan 31056, Korea; sugug12@kitech.re.kr (G.-S.C.); binufamily@kitech.re.kr (H.-W.P.); 2 Department of Green Process and System Engineering, University of Science & Technology (UST), Daejeon 34113, Korea; 3 Department of Chemical and Biological Engineering, Korea University, Seoul 02841, Korea; leejhyyy@korea.ac.kr

**Keywords:** epoxy toughening, phenol-terminated polyurethane, carboxyl-terminated butadiene acrylonitrile copolymer, impact wedge-peel test, structural adhesive

## Abstract

Epoxy adhesives are widely used in various industries because of their high heat and chemical resistance, high cohesion, and minimal shrinkage. Recently, epoxy adhesives have been applied in the automotive industry as structural adhesives for lightweight vehicles. However, the brittleness of the epoxy is an obstacle for this application, since the automotive industry requires epoxy-based structural adhesives to have a high level of high-speed impact resistance. Hence, we used phenol-terminated polyurethane (PTPU) as a toughening agent for epoxy adhesives and compared the results with those that were obtained with carboxyl-terminated butadiene acrylonitrile copolymer (CTBN). The high-energy impact resistance of the epoxy adhesives was measured by the impact wedge-peel (IWP) test, and the shear strength was measured by the single lap joint test. As a result, the 20 wt % PTPU-modified epoxy adhesive showed remarkably higher total absorbed energy (25.8 J) during the IWP test and shear strength (32.3 MPa) as compared with the control epoxy adhesive (4.1 J and 20.6 MPa, respectively). In particular, the total absorbed energy of the PTPU-modified epoxy adhesive was much larger than that of the CTBN-modified epoxy adhesive (5.8 J). When more than 10 wt % PTPU was added, the modified epoxy adhesives showed stable crack growth and effectively transferred external stress to the substrate. These results were explained by changes in the glass transition temperature, crosslinking density, and morphology due to the toughening agents.

## 1. Introduction

Structural adhesives are used for structural bonding that requires high adhesion strength, elasticity, and environmental resistance. Particularly, structural adhesives are emerging as a fastening method for replacing bolts and nuts in the field of transportation vehicles in order to reduce carbon emissions and energy consumption. To be used as a structural adhesive for automobiles, high shear strength as well as high peel, bending, impact, and fatigue strengths are required [1]. Therefore, structural adhesives for automobiles are commonly prepared with epoxy and polyurethane [2,3]. Epoxy-based structural adhesives are the most suitable structural adhesives for fastening parts that require high-speed impact resistance, because they have high cohesive strength, low shrinkage, and high heat resistance and chemical resistance. In addition, epoxy-based structural adhesives can provide various formulations that are suitable for bonding processes and required properties by adjusting the curing agent and additives [4]. However, despite these advantages, the brittleness of epoxy resin needs to be improved in order to achieve the impact resistance required of a structural adhesive [5].

It is common to introduce core-shell rubber (CSR) particles or soft toughening agents to improve the toughness of epoxy resins. Butadiene acrylonitrile (BN) liquid rubbers are the most prevalent compounds used as toughening agents [6,7,8,9]. BN liquid rubbers are classified by their terminal groups; BN liquid rubbers have amine, carboxylic acid, and epoxy at their ends and they are named ATBN, CTBN, and ETBN, respectively [10,11,12,13,14,15,16,17,18,19,20,21,22,23]. These groups can participate in the curing reaction. These reactive liquid rubbers form a homogeneous phase when mixed with an epoxy resin but are phase-separated during the curing reaction to form a sea-island structure; this sea-island structure is very important for improving epoxy toughness [24]. In particular, the toughness improvement of epoxy resins using CTBN have been studied from various viewpoints as follows.

Kunz-Douglass et al. used CTBNs with different acrylonitrile contents (0%, 10%, 18%, and 27%) to know the relationship between the CTBN particle size and epoxy toughness [25]. According to their report, as the acrylonitrile content increased, the rubber particle size decreased, and the tear strain increased. The fracture toughness of the epoxy resin was highly dependent on the volume fraction of the CTBN, but less dependent on the particle size. Hsu and Liang prepared CTBN-modified epoxy resins and studied the toughness enhancement by the interpenetrating network (IPN), which were developed by benzoyl peroxide (BPO) and dicumyl peroxide (DCP) [26]. According to their report, fracture toughness was significantly improved by IPN structure. The epoxy system with 3 wt % BPO showed about 70% improved fracture toughness as compared to the system without IPN structure. Recently, Bach et al. reported that they produced CTBN-grafted epoxdized soybean oil (ESO-*g*-CTBN) by ring-opening reaction between epoxide group of soybean oil and carboxyl group of CTBN to improve the fracture toughness and mechanical properties of the epoxy [27]. According to their results, the fracture toughness of 15 part per hundred resin (phr) ESO-*g*-CTBN modified epoxy resin was improved by 67.7% when compared to neat epoxy through the reduction of *T*_g_ and crosslink density.

In addition, thermoplastics with a low glass-transition temperature (*T*_g_) and reactive groups are also used as toughening agents. For example, thermoplastic polyurethane (TPU), which exhibit various properties depending on the polyols, are also used as toughening agents for epoxy resins [28,29,30,31]. As a toughening agent, TPU should be elastic at room temperature, so it has a low *T_g_* like CTBN; the *T*_g_ of TPU is known to have a value of −80 to−40 °C, depending on the type of polyols [29,30]. In addition, similar to the CTBN cases, the functionalized TPU forms a dispersed phase by the reaction-induced phase separation as the curing reaction proceeds, and induces a strong chemical bond at the TPU domain/epoxy matrix interface [29]. It is also known that the crosslink density, *T*_g_, and toughness of the TPU/epoxy blends depend largely on their final morphologies, which, in turn, are determined by the phase separation [31,32]. In this regard, epoxy toughening studies using various TPUs have been conducted, as follows.

Wang and Chen synthesized phenolic hydroxyl-terminated polyurethane (HTPU) and aromatic amine-terminated polyurethane with different macroglycols and evaluated their toughening effect after blending with epoxy [29]. According to their report, the fracture energy of epoxy increased 10 times with 15 phr HTPU when compared to neat epoxy. Bhuniya and Adhikari used hydroxy-terminated silicon modified polyurethane (SiMPU) oligomers to increase the fracture toughness of epoxy resins [30]. With 20 phr SiMPU, the fracture toughness increased five times as compared to neat epoxy. Recently, Zou et al. studied changes in toughness according to the type and content of hyperbranched polyurethane (HBPU) and linear analog polyurethane (LPU) when they were used as epoxy toughening agents [31]. According to the report, HBPU had better miscibility with epoxy resin than LPU, and 10 wt % HBPU improved toughness three times over neat epoxy.

On the other hand, a typical method for measuring the impact resistance of an epoxy resin is the Izod or Charpy impact test, which applies a relatively low-speed impact [33,34,35,36,37]. However, this method is not suitable for structural adhesives for automobiles that are exposed to high-speed impact energies. For this reason, the impact wedge-peel test (IWP test) was developed in order to evaluate the fracture behaviour of adhesive joints in response to high-speed impacts and was adopted as an international standard (ISO 11343) test method by the automotive industry in 1993 [38]. There have been several studies on epoxy-based structural adhesives while using the IWP test method [39,40,41]. Blackman et al. were the first to report the measurement of the resistance to cleavage fracture of a structural adhesive by the IWP test; these authors measured the fracture energy through a T-peel test and comparing it with the IWP test result [39]. Later, Taylor et al. evaluated the performance of commercially available epoxy-based structural adhesives using the IWP test method [40]. Although Back et al. recently used the IWP test to measure the impact strength of form-type epoxy adhesives containing CSR and analysed the effect of the degree of foaming, there are few studies on the effect of thermoplastic polyurethanes and reactive liquid rubbers on the impact resistance of epoxy-based structural adhesives using the IWP test [41].

Therefore, in this study, phenol terminated polyurethane (PTPU), a thermoplastic polyurethane, and carboxyl-terminated butadiene acrylonitrile copolymer (CTBN), a reactive liquid rubber, were used as toughening agents for structural adhesives prepared with diglycidyl ether of bisphenol-A (DGEBA) epoxy as a main component, and their effects on the physical properties of the structural adhesives were examined according to their content. The resistance to cleavage fracture, an index of impact resistance, was determined by the IWP test using CR340, which is used for automobile pillars [42], and the adhesive strength was measured by the single lap shear test. To analyse the results of the IWP and lap shear test, changes in the *T*_g_, crosslinking density and viscosity of the epoxy adhesives according to the addition of PTPU and CTBN were measured, and fracture surface and surface morphology analyses were performed in this study.

## 2. Experiments

### 2.1. Materials

**Figure 1:**
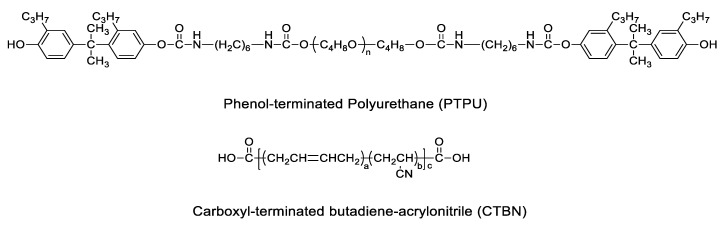
Chemical structures of phenol-terminated polyurethane (PTPU) and carboxyl-terminated butadiene acrylonitrile (CTBN).

The bisphenol-A type of epoxy resin (DGEBA, diglycidyl epoxy of bisphenol A, YD-128), the carboxyl-terminated butadiene copolymer with 26% acrylonitrile (Hypro 1300X13 CTBN), and the dimer acid-modified epoxy resin used for diluent (YD-171) were purchased from Kukdo Chemical Company (Seoul, South Korea). Phenol-terminated polyurethane (DY-965) was purchased from Huntsman Corporation (USA). A 40 wt % core-shell rubber (CSR) dispersed in DGEBA epoxy resin (MX-154) was purchased from Kaneka Corporation (Tokyo, Japan). Dicyandiamide hardener (Dyhard 100) and *N*,*N*-dimethyl-*N*-phenylurea accelerator (Omicure U-405) were purchased from AlzChem Group (Trostberg, Germany) and DyStar Group (Singapore, Singapore), respectively. For the fillers, calcium carbonate (Omyacarb 10) was used and supplied by OMYA Company (Oftringen, Switzerland). Additionally, glass beads with a diameter of 0.2 mm to control the thickness of the adhesive were supplied by UNITECH (Ansan, South Korea). Figure 1 shows the chemical structures of PTPU and CTBN, and Table 1 shows the details of the materials used for the epoxy adhesive.

### 2.2. Preparation of Epoxy Adhesive

Table 2 shows the epoxy adhesive composition according to the PTPU or CTBN content. First, PTPU or CTBN was poured in the prepared container at 0, 5, 10, 15, 20, and 30 wt % according to the composition and, then, the CSR mixture was added. Additionally, DGEBA was added to adjust the epoxy ratio to the epoxy resin contained in the CSR mixture. Before adding the curing agent, the materials were blended and defoamed in a paste mixer (ARV-310, Thinky, Tokyo, Japan) at 2000 rpm under 1.0 kPa vacuum condition for three minutes. When the mixing was complete, DICY, accelerator, and CaCO_3_ were added and mixed in the paste mixer at 2000 rpm for three minutes. Finally, 1 wt % DAME by weight was added, mixed in the paste mixer, and defoamed at 2000 rpm under 1.0 kPa vacuum condition for three minutes.

### 2.3. Preparation of the Impact Wedge-Peel (IWP) Test and Single Lap Joint (Lap Shear) Test Specimens

**Figure 2:**
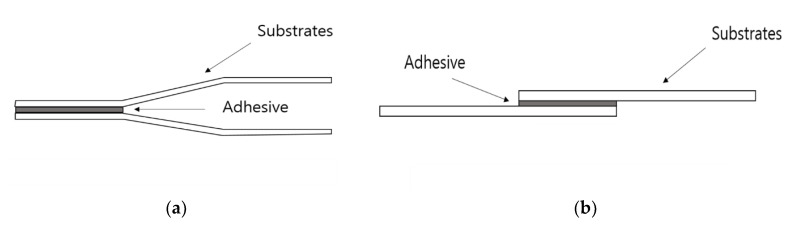
Applied adhesive on (**a**) the impact wedge-peel (IWP) test specimen; and (**b**) the single lap joint (lap shear) test specimen.

The ISO 11343 Standard does not specify the dimensions to be used for the IWP specimens. Hence, we adopted the widely used Ford specimen design (length: 90 mm, width: 20 mm, thickness: 1.6 mm, material: CR340). Two specimens were prepared and the surface of each specimen was wiped with acetone to remove dust. The mixture was applied to an area of 30 mm × 20 mm on the specimen surface, and a small amount of glass beads was spread onto the well-coated mixture. Each specimen was nested, as shown in Figure 2a, and cured for 28 min. at 180 °C in a conventional oven.

We used a specific lap shear specimen to prepare for the single lap joint (lap shear) test, as described in the ISO 4587 Standard (length: 100 mm, width: 25 mm, thickness: 1.6 mm, material: CR340) [43]. In the same manner as the IWP test, two specimens were prepared, and the surface of each specimen was wiped with acetone to remove dust. The epoxy mixture was applied to an area of 25 mm × 12.5 mm on the specimen surface, and a small amount of glass beads, which controlled the thickness of applied adhesive, was spread onto the well-coated mixture. Each specimen was overlaid, as shown in Figure 2b, and then cured for 28 min. at 180 °C in a conventional oven.

### 2.4. Preparation of Dynamic Mechanical Analyser (DMA) Specimens

DMA specimens were prepared by casting the epoxy adhesives in silicone mould. The rectangular specimens (length: 32 mm, width: 2.0 mm, thickness: 1.0 mm) were employed for DMA measurement. The epoxy adhesive mixtures were poured into the silicone mould and degassed for an hour in an 80 °C vacuum oven and cured 28 min. in a 180 °C conventional oven. After curing, the specimens were cooled down to room temperature and separated from the silicone mould.

### 2.5. Characterizations

The viscosity of the epoxy mixture was measured by a Brookfield DV1 viscometer (AMETEK Brookfield, Middleborough, MA, USA). The viscosity measurements were conducted at room temperature while using a HB-07 spindle at 2.0 rpm.

The IWP tests of the epoxy adhesive test specimens were performed using an impact drop tower (Model 7520, Instron, Norwood, MA, USA) at room temperature. Two bent steel plates were bonded using the epoxy adhesives (area: 30 mm × 20 mm, thickness: 0.2 mm), and the force was measured when the adhesive layer was cleaved by the wedge at a velocity (v) of 2.0 m/s, as per ISO 11343. The cross-section morphologies of the epoxy adhesives after the IWP tests were observed by FE-SEM (JSM 6701F, JEOL, Tokyo, Japan).

The single lap shear tests of the epoxy adhesive test specimens were conducted using a dual-column universal testing machine (Model 5969, Instron, Norwood, MA, USA). The crossed specimens were bonded using the epoxy adhesives (area: 25 mm × 12.5 mm, thickness: 0.2 mm), and the shear stress measurements were performed at room temperature at 5 mm/min. crosshead displacement, as per the ISO 4587 standard.

The crosslink density (*ρ*) and glass transition temperature (*T*_g_) of the epoxy adhesives were measured by a dynamic mechanical analyser (DMA 8000, Perkin Elmer, Waltham, MA, USA). The rectangular specimens (length: 32 mm, width: 2.0 mm, thickness: 1.0 mm) were prepared and tested in tension mode from −100 to 250 °C with 5 °C/min. ramp rate at a frequency of 1 Hz.

The molecular weights of PTPU and CTBN were measured by gel-permeation chromatography (GPC) (P-4000, FUTECS, Daejeon, South Korea). As a control, polystyrene with a PDI of approximately 1 was prepared. Subsequently, the sample was dissolved in THF for HPLC at 1 wt %. After that, the solution was placed in a cylinder and filtered by a syringe filter with 0.2 μm pore size. Finally, each sample was injected into GPC and then measured at 40 °C.

## 3. Results and Discussion

### 3.1. Thermomechanical Properties of the Epoxy Adhesives

**Figure 3:**
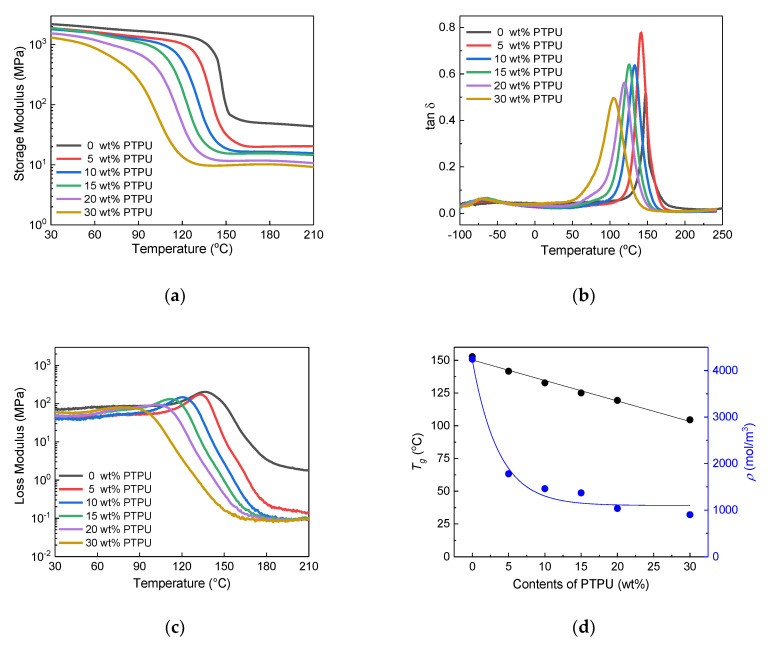
Storage modulus (**a**); loss tan δ (**b**); and loss modulus (**c**) curves of the PTPU-toughened epoxy adhesives and their *T*_g_ and crosslink densities (**d**) as a function of the PTPU content.

The glass transition temperature (*T*_g_) and crosslink density (*ρ*) of the cured epoxy adhesives were measured by a dynamic mechanical analyser (DMA) in order to determine the effect of the toughening agents. Figure 3 shows the storage modulus, loss tan δ, loss modulus, *T*_g_s and crosslink density of the epoxy adhesives as a function of the PTPU content. The glass-transition temperature was determined by the peak value of the tan δ curves, and the crosslink density was calculated based on the rubber elasticity theory [44,45]. As expected, the *T*_g_ and storage modulus of the epoxy adhesives decreased as the PTPU content increased. Figure 3d shows that the *T*_g_ decreased by approximately 8 °C for every 5 wt % increase in PTPU. The crosslink density remarkably decreased after the initial addition of PTPU, but the extent of decrease after the further addition of PTPU was diminished.

FT-IR analysis of PTPU heat-treated at 180 °C was conducted to observe whether the urethane groups in PTPU reverted to isocyanate and alcohol to form allophanates during curing reaction. The reverse reaction scarcely occurred under our curing condition, and allophanate formation was too trivial, as shown in Figure S1.

**Figure 4:**
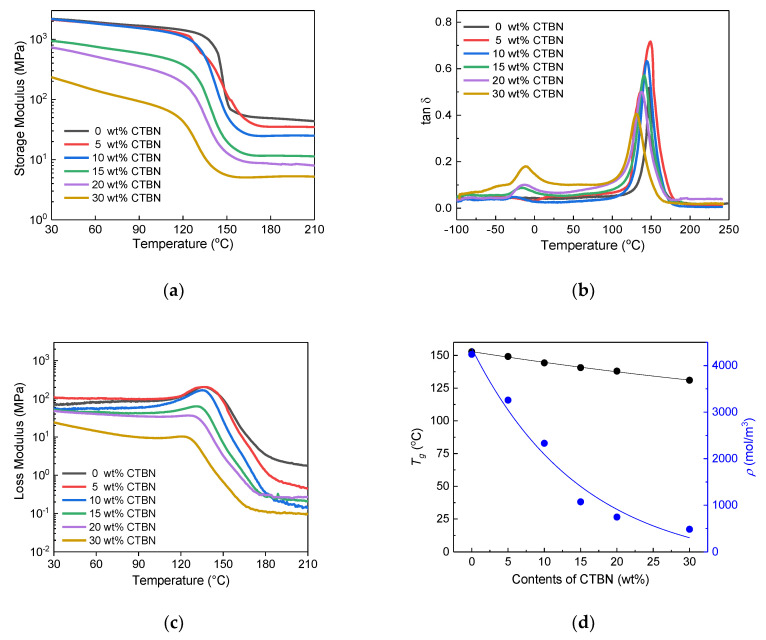
Storage modulus (**a**); loss tan δ (**b**); and loss modulus (**c**) curves of the CTBN-toughened epoxy adhesives and their *T_g_* and crosslink densities (**d**) as a function of the CTBN content.

CTBN led to results that were similar to those of PTPU, as shown in Figure 4. However, the *T*_g_ decreased by 4 °C for every 5 wt % because of the relatively high *T_g_* of CTBN (−12 °C vs. −70 °C for PTPU). The crosslink density was also changed in a manner that differed from that of PTPU; it was constantly decreased with the CTBN content. When CTBN exceeded 15 wt %, the crosslink density became lower than that of the PTPU-modified sample. This result seemed to be due to the differences in the size, miscibility, and functional groups of CTBN and PTPU.

The GPC results show that CTBN has a much lower molecular weight (*M_n_*: 6800) than PTPU (*M_n_*: 18,700). CTBN may be more uniformly distributed in the epoxy network, causing a reduced crosslink density as compared with that caused by PTPU, owing to its small size and high miscibility. This observation is supported by the CTBN domain size, which is relatively smaller than that of PTPU. On the other hand, the reduced crosslink density of the PTPU-toughened epoxy adhesives could be explained, as follows. Based on the crosslink density and morphology of the PTPU-toughened epoxy adhesives, the sharp decrease in the crosslink density of the PTPU-toughened epoxy (at 5 wt % of PTPU) was believed to result from the hindrance of the curing reaction by the PTPU domains. However, as the PTPU content increased, more PTPU-epoxy adducts were formed and included in the network structure, alleviating the decrease in the crosslink density. In conclusion, it is believed that the crosslink density is determined by the degree of phase separation and the ease of toughening agent-epoxy adduct formation.

**Figure 5:**
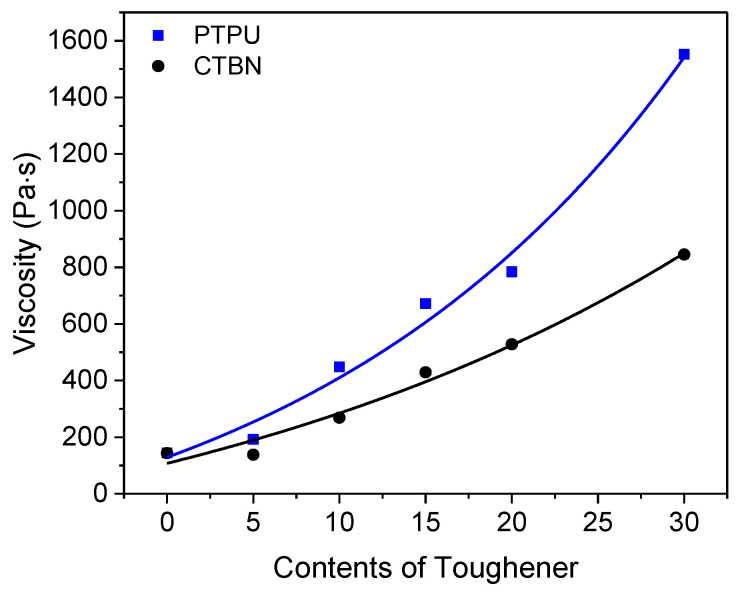
Change in the viscosity of the epoxy adhesives by the addition of PTPU and CTBN.

Viscosity is also an important physical property of epoxy adhesives. Normally viscosity increases with the addition of PTPU or CTBN [46]. As shown in Figure 5, the viscosity of the PTPU-modified epoxy adhesive was twice as high as that of the CTBN-modified epoxy adhesive, because PTPU has higher viscosity than CTBN. Because too-high viscosity causes a wetting problem, more than 30 wt % of PTPU could not be used.

### 3.2. Impact Wedge-Peel (IWP) Properties

**Figure 6:**
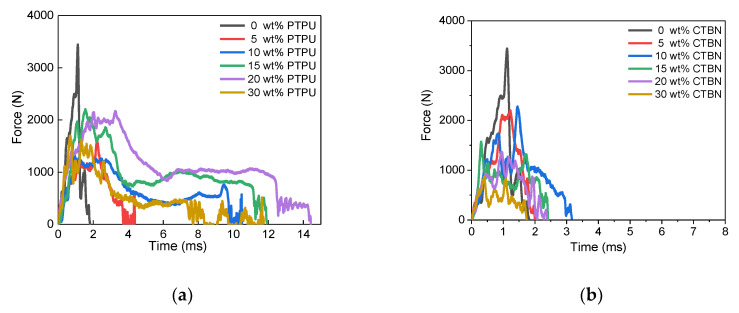
Time-to-force curves of the epoxy adhesives toughened with PTPU (**a**) and CTBN (**b**) measured by the IWP test method.

The dynamic mechanical properties of the epoxy adhesives were measured by the IWP test, which is a particularly easy method for measuring the resistance to cleavage fracture. Figure 6 shows the time-to-force curves obtained from the IWP tests of the PTPU- and CTBN-toughened epoxy adhesives. According to Blackman et al., the case in which the force value is not immediately broken after the impact and has a plateau region between 25% and 90% of the measurement time is called “stable crack growth.” In contrast, the case in which the adhesive force cannot be sustained and is broken after the impact is called “unstable crack growth” [38].

In Figure 6, the PTPU-toughened epoxy showed a plateau region when 10 wt % or more PTPU content was added. However, the CTBN-toughened epoxy adhesives showed no plateau region, regardless of the CTBN content, meaning that all of the CTBN-toughened epoxies were broken by unstable crack growth. Based on the IWP test results, the dynamic resistance to cleavage, cleavage-force, displacement for cleavage, and total absorbed energy were obtained, as follows. The dynamic resistance to cleavage is the average value of force in the plateau region, and the cleavage-force is the average value of force measured between 25% and 90% of the measurement time. The displacement for cleavage is obtained by multiplying the measurement time and test speed (2 m/s), and the total absorbed energy is calculated by multiplying the area of force-to-time curve and test speed.

**Figure 7:**
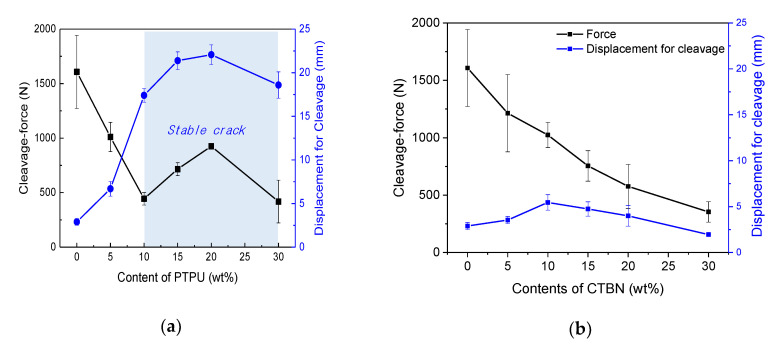
Cleavage-force (■) and displacement for cleavage (●) of the PTPU-toughened (**a**); and CTBN-toughened (**b**) epoxy adhesives.

**Figure 8:**
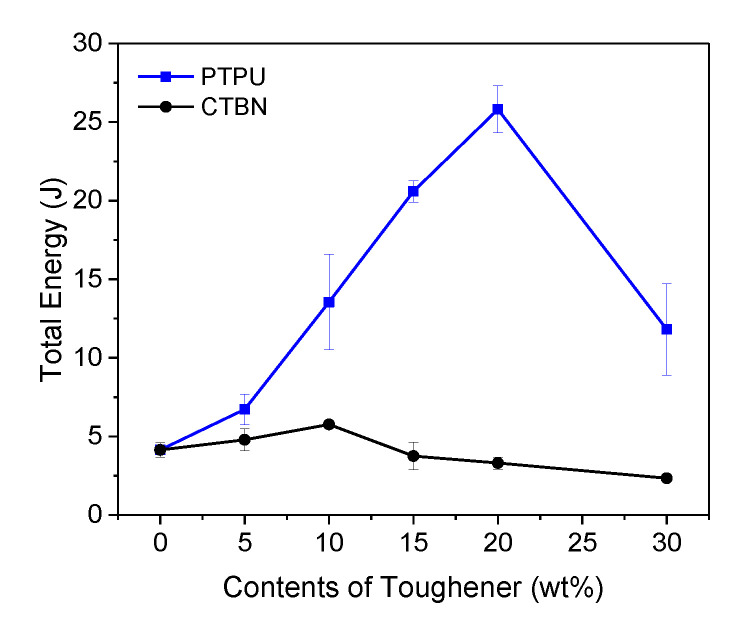
Total absorbed energy of the PTPU- and CTBN-toughened epoxy adhesives as a function of toughening agent contents.

Figure 7 shows the cleavage-force and displacement for the cleavage of the PTPU- and CTBN-toughened epoxy adhesives, and Figure 8 shows the total absorbed energy of the PTPU- and CTBN-toughened epoxy adhesives. Figure 7 shows that the cleavage-force values of 0 and 5 wt % PTPU contents were greatly decreased. The higher the PTPU content, the higher the cleavage-force value in the stable crack growth region. However, the stable and unstable crack growth coexisted in the 30 wt % PTPU samples. The displacement for cleavage increased with the PTPU content, especially in the stable crack growth region, and then decreased in the 30 wt % PTPU samples, like the cleavage-force value. Moreover, the cleavage-force of the CTBN-toughened epoxy adhesives decreased monotonically with the CTBN content, and the displacement for cleavage seemed to increase and then decrease with the CTBN content, but the difference was trivial.

In the case of the total absorbed energy, the PTPU-toughened epoxy adhesives exhibited much higher values than the CTBN-toughened epoxy adhesives for all of the compositions. In addition, the PTPU-toughened epoxy adhesives showed noticeable changes in the total absorbed energy with the PTPU content. However, the CTBN-toughened samples showed almost constant values, regardless of the content. Thus, the CTBN-toughened epoxy adhesives are not tough enough to endure the high-speed impact energy delivered by the IWP test. When considering Figure 7, this difference seems to originate mainly from the displacement for cleavage. To explain this, the deformed IWP test specimens and their fracture surfaces were examined.

**Figure 9:**
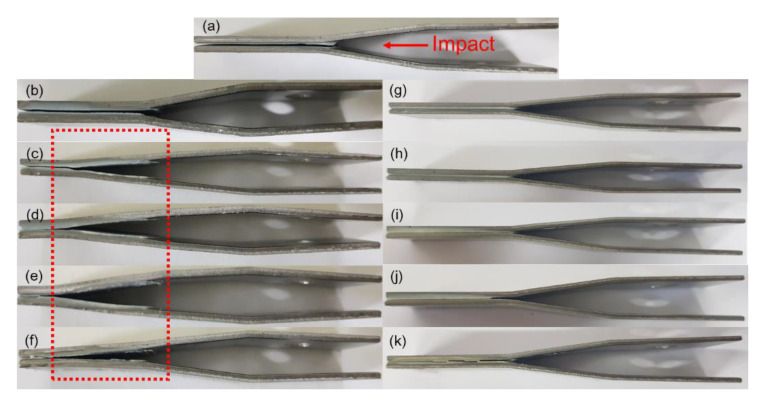
Photos of specimens after the IWP test: (**a**) control; (**b**) 5 wt % PTPU; (**c**) 10 wt % PTPU; (**d**) 15 wt % PTPU; (**e**) 20 wt % PTPU; (**f**) 30 wt % PTPU; (**g**) 5 wt % CTBN; (**h**) 10 wt % CTBN; (**i**) 15 wt % CTBN; (**j**) 20 wt % CTBN; and, (**k**) 30 wt % CTBN.

Figure 9 shows the deformed specimens after the IWP test. The specimens showing large total absorbed energies were severely deformed in the adhesive layers (Figure 9c–f)) and had a plateau region of stable crack growth. Thus, the more external impact energy that was transferred to the specimen, the higher the absorbed energy. Therefore, not only the toughness of the adhesive but also the interfacial adhesion is very important for achieving large absorbed energy.

It is necessary to introduce a soft material into the crosslinking structure or to complicate the crack propagation pathway through the formation of separate domains in order to improve the toughness of the epoxy. Due to this soft material, the *T_g_* and crosslink density of the epoxy were reduced. It is also known that excessive reduction in the crosslink density leads to a decrease in toughness [47]. When considering Figure 3d and Figure 4d, at 10 wt % of toughening agent, the PTPU-toughened epoxy adhesive seems to be softer than the CTBN-toughened epoxy adhesives based on their *T_g_* and crosslink densities. As a result, the PTPU-toughened epoxy has higher total absorbed energy than the CTBN-toughened epoxy. As mentioned in 3.1, the PTPU-toughened epoxy adhesives showed a trivial reduction in crosslink density, while their *T_g_* significantly decreased with further increases in PTPU. However, the CTBN-toughened epoxy adhesives showed a serious decrease in crosslink density, which became lower than that of the PTPU-toughened epoxy adhesives after 15 wt % CTBN was added. This result implies that a high content of CTBN reduced the fracture toughness of the epoxy adhesives by an excessive reduction in crosslink density.

**Figure 10:**
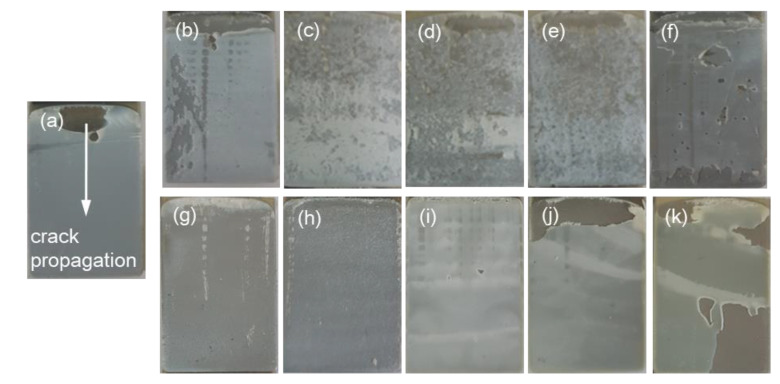
Fracture surfaces of IWP specimens: (**a**) control; (**b**) 5 wt % PTPU; (**c**) 10 wt % PTPU; (**d**) 15 wt % PTPU; (**e**) 20 wt % PTPU; (**f**) 30 wt % PTPU; (**g**) 5 wt % CTBN; (**h**) 10 wt % CTBN; (**i**) 15 wt % CTBN; (**j**) 20 wt % CTBN; and, (**k**) 30 wt % CTBN.

Based on the fracture surface analysis, most of the samples showed cohesive failure of the epoxy adhesives, except for the samples in Figure 10j,k, which showed interfacial failure. It is difficult to distinguish between interfacial and cohesive failures because for some specimens, the adhesive at the impact site was broken off from the surface due to the high-impact energy. However, when considering the fracture surfaces after the lap shear test, we know that epoxy adhesives that contain more than 15 wt % CTBN have weak interfacial adhesion. This weak interfacial adhesion is because the amount of epoxy in the adhesives decreases as the content of CTBN increases. This interfacial failure is more easily observed in the CTBN-toughened epoxy adhesives, because CTBN is smaller than PTPU (*M_n_* = 6800 vs. 18,700). Therefore, epoxy adhesives have three times more CTBN molecules than PTPU molecules, even at the same wt %.

### 3.3. Fracture Surface Morphology of IWP Specimens

It is well known that the separate domain formed by the toughening agent during the curing reaction plays an important role in improving the toughness of the epoxy. The number and size of the domain depend on the solubility parameter and chemical interaction between the toughening agent and epoxy [48,49,50].

**Figure 11:**
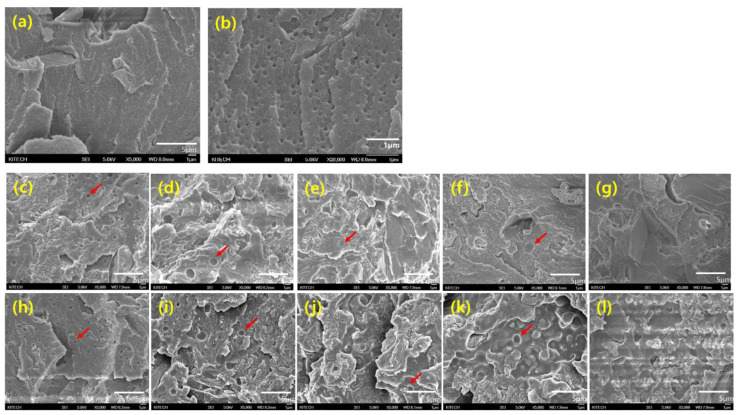
SEM micrographs of the fractured surfaces of the control and modified epoxy adhesives obtained from the IWP test (5000× magnification except (**b**)): (**a**) control; (**b**) control (20,000× magnification); (**c**) 5 wt % PTPU; (**d**) 10 wt % PTPU; (**e**) 15 wt % PTPU; (**f**) 20 wt % PTPU; (**g**) 30 wt % PTPU; (**h**) 5 wt % CTBN; (**i**) 10 wt % CTBN; (**j**) 15 wt % CTBN; (**k**) 20 wt % CTBN; and, (**l**) 30 wt % CTBN.

Figure 11 shows the fractured surface morphology of the epoxy adhesives that were modified with different amounts of PTPU or CTBN. In the control sample, 200–300-nm spherical domains and 12-µm irregular-shaped particles are observed due to the CSR and CaCO3 particles (Figure 11a,b). These domains and particles are observed in all of the samples in Figure 11. After adding the PTPU, 1-µm spherical domains appeared, and their size increased with the addition of up to 10 wt % PTPU (see Figure 11c,d). However, as shown in Figure 11e,f, the number and size of the spherical domains decreased rapidly, and they are not observed in Figure 11g. From Figure 11h–l, the CTBN-toughened epoxy adhesives show similar morphology to the PTPU-toughened epoxy adhesives. However, CTBN developed slightly smaller domains than PTPU.

### 3.4. Single Lap Shear Properties

**Figure 12:**
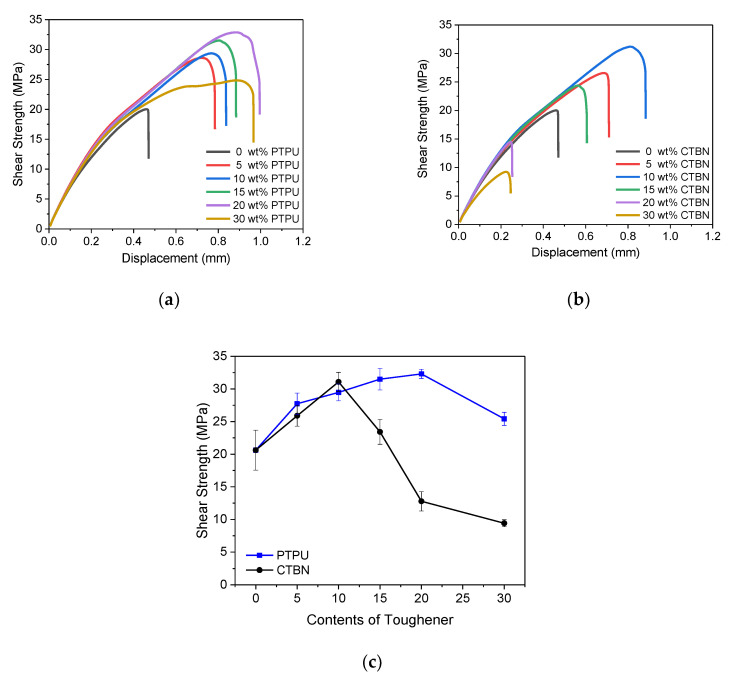
Shear strength-displacement curves of the PTPU- (**a**); and CTBN- (**b**) toughened epoxy adhesives, and comparison of the shear strength of the epoxy adhesives with respect to the toughening agent; and its content (**c**).

**Figure 13:**
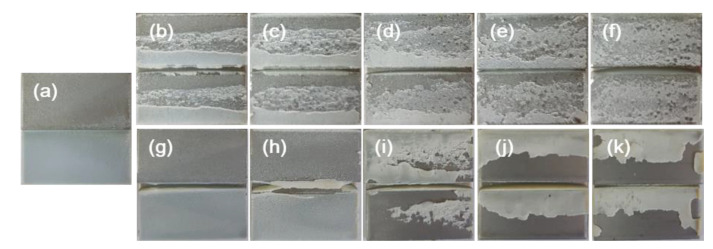
Fracture surfaces of epoxy adhesives obtained by lap shear test: (**a**) control; (**b**) 5 wt % PTPU; (**c**) 10 wt % PTPU; (**d**) 15 wt % PTPU; (**e**) 20 wt % PTPU; (**f**) 30 wt % PTPU; (**g**) 5 wt % CTBN; (**h**) 10 wt % CTBN; (**i**) 15 wt % CTBN; (**j**) 20 wt % CTBN; and, (**k**) 30 wt % CTBN.

The shear strength of the epoxy adhesives was measured and is shown in Figure 12. When PTPU was used, the shear strength and displacement of the epoxy adhesives increased with the PTPU content due to the increased toughness. In particular, the failure mode changed from brittle to ductile at 30 wt % PTPU, where plastic deformation was clearly observed. However, all of the CTBN-toughened epoxy adhesives were fractured in brittle mode and their shear strength and displacement decreased when the CTBN content was over 15 wt %. In particular, clear substrate was observed in the fracture surfaces taken from the 15 wt % or more CTBN-toughened epoxy adhesives (see Figure 13). After examining the fracture surface and *T*_g_ and *ρ* values, the drastic decrease in the shear strength of the CTBN-toughened epoxy adhesives is attributed to a decrease in the interfacial adhesion and the crosslink density.

## 4. Conclusions

Polyurethane (PTPU)- and butadiene-acrylonitrile rubber (CTBN)- based toughening agents were used to improve the high-speed impact resistance of epoxy-based adhesives, and the following conclusions were obtained.

The glass-transition temperatures and crosslink densities of the epoxy adhesives changed differently, depending on the toughening agent (PTPU or CTBN). The degree of *T*_g_ reduction was highly dependent on the *T*_g_ of the toughening agent itself, and the crosslink density depends on the phase separation and the ease of formation of toughening agent-epoxy adducts.

From the results of the IWP test, stable crack growth is observed if the epoxy adhesive effectively transfers external stress to the substrate, which means that the adhesive strength with the substrate and the toughness of the adhesive are very important. PTPU has very good elongation and recovery properties, showing excellent effects on improving toughness, and the urethane group is a site for H-bonding for the substrate.

The IWP test does not show meaningful results in the case of CTBN, which is known to have a toughness improving effect. This finding suggests that the mechanism for improving toughness by CTBN is not effective for high-energy impacts due to the decrease in the crosslink density and interfacial adhesion.

## Figures and Tables

**Table 1:** Materials used for the epoxy adhesives.

Materials	Composition	Abbreviation	Equivalent Weight (g/eq)
Epoxy	Diglycidyl epoxy of bisphenol A	DGEBA	187
Diluent agent	Dimer acid modified epoxy	DAME	430
Curing agent	Dicyandiamide	DICY	21.02
	*N*,*N*-dimethyl-N-phenyl urea	Accelerator	3
CSR + Epoxy	CSR in epoxy resin (40 wt %)	CSR mixture	301

**Table 2:** Composition of epoxy adhesives.

Sample	DGEBA (g)	Toughening Agent (g)	CSR Mixture (g)	DICY (g)	Accelerator (g)	CaCO_3_ (g)
Control	30	0	15	3.12	0.39	14
5 wt % Toughening agent		3.29				
10 wt % Toughening agent		6.95				
15 wt % Toughening agent		11.03				
20 wt % Toughening agent		15.63				
30 wt % Toughening agent		26.79				

* All of the samples were added with 1 wt % DAME by total sample weight.

## Supplementary Materials

The following are available online at https://www.mdpi.com/2073-4360/12/7/1549/s1. FT-IR analysis, Figure S1: FT-IR spectra of pristine and 180 °C heat treated PTPUs (a), NCO stretching at 2270 cm⁻^1^ (b), and fingerprint region (1800 to 1150 cm⁻^1^) (c), Table S1: Comparison of differences in FT-IR spectra.
