# Supplementary material for: Comparative Study on the Impact Wedge-Peel Performance of Epoxy-Based Structural Adhesives Modified with Different Toughening Agents
Source: Polymers (Basel). 2020 Jul 13;12(7):1549. doi: 10.3390/polym12071549 (PMC7408613; doi:10.3390/polym12071549)
Supplement: Supplementary file 1 [file polymers-12-01549-s001.pdf]

Supplement Information

Comparative study on the impact wedge-peel performance of epoxy-based structural adhesives modified with different toughening agents

Gyeong-seok Chae [1,2], Hee-woong Park [1,3], Jung-Hyun Lee [3], Seunghan Shin [1,2,*]

FT-IR analysis: The chemical structure changes of PTPU by heat treatment were analysed using FT-IR spectrometer. Infrared spectra were recorded using the KBr window technique on a FT-IR (Nicolet 6700, Thermo Fisher Scientific, USA) spectrometer from 4000 to 1000 cm^{-1} with 16 scans and 4 cm^{-1} resolution. Small amount of viscous liquid PTPU was bar coated on the KBr window (diameter: 25 mm, thickness: 4 mm) and its IR spectra were recorded before and after heat treatment at 180 °C for 28 min.

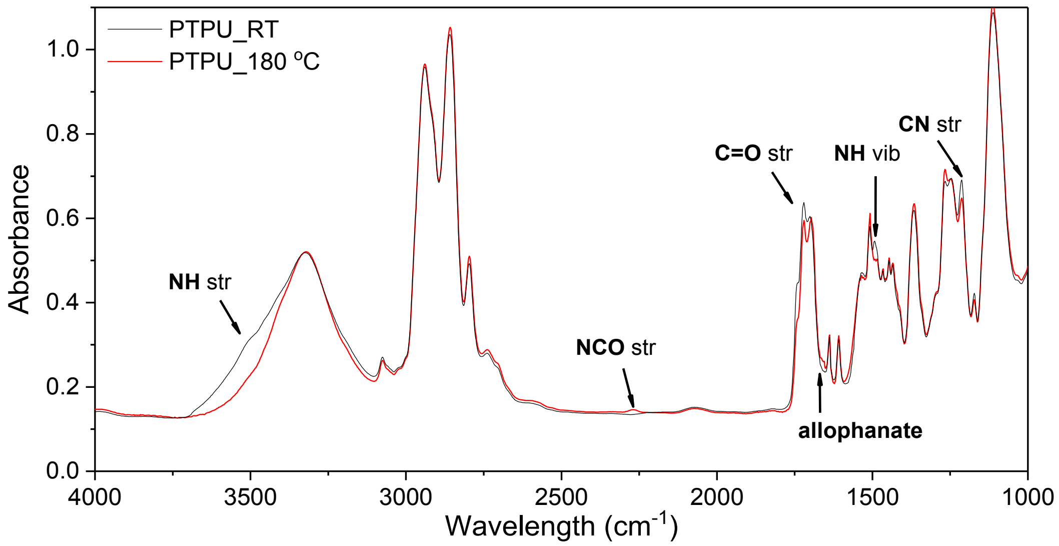

(a)

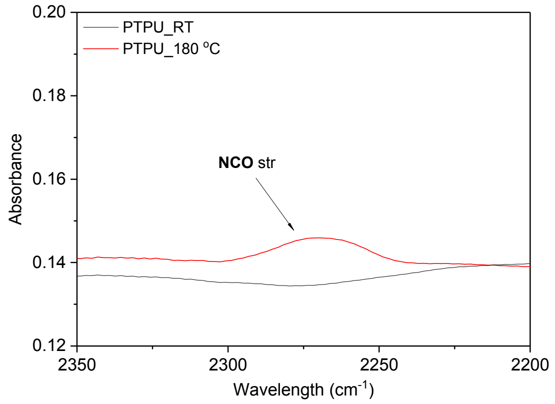

(b)

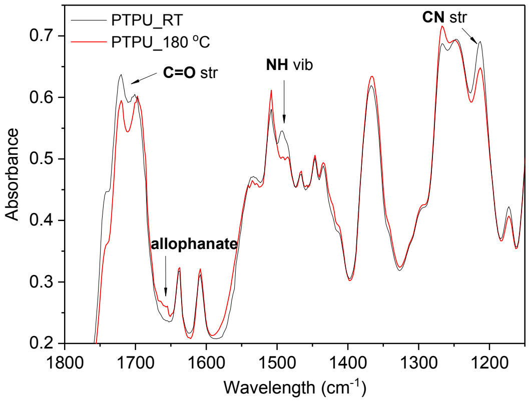

(c)

Figure S1. FT-IR spectra of pristine and 180 °C heat treated PTPUs (**a**), NCO stretching at 2270 cm^{-1} (**b**), and fingerprint region (1800 cm^{-1} to 1150 cm^{-1}) (**c**).

Table S1. Comparison of differences in FT-IR spectra

Wavenumber (cm^{-1})	Vibration type	Assignment	ref
1213	stretching	urethane C-N	[2]
1490	vibrating	urethane N-H	[2]
1650 - 1668	stretching	allophanate C=O	[3]
1700	stretching	urethane C=O	[1]
2270	stretching	NCO	[1]

Reference:

1. Kothandaraman, H.; Nasar, A.S. The thermal dissociation of phenol-blocked toluene diisocyanate crosslinkers. *J. Macromol. Sci. Part A* **1995**, doi:10.1080/10601329508009343.

2. Asefnejad, A.; Khorasani, M.T.; Behnamghader, A.; Farsadzadeh, B.; Bonakdar, S. Manufacturing of biodegradable polyurethane scaffolds based on polycaprolactone using a phase separation method: physical properties and in vitro assay. *Int. J. Nanomedicine* **2011**, doi:10.2147/ijn.s15586.

3. Ramakrishna, S.; Kumar, K.S.S.; Mathew, D.; Nair, C.P.R. Long-living, stress- and pH-tolerant superhydrophobic silica particles via fast and efficient urethane chemistry; Facile preparation of self-recoverable SH coatings. *J. Mater. Chem. A* **2015**, doi:10.1039/c4ta04901b.
